# Association between single nucleotide polymorphisms (SNPs) of *XRCC2* and *XRCC3* homologous recombination repair genes and triple-negative breast cancer in Polish women

**DOI:** 10.1007/s10238-014-0284-7

**Published:** 2014-04-13

**Authors:** Beata Smolarz, Marianna Makowska, Dariusz Samulak, Magdalena M. Michalska, Ewa Mojs, Maciej Wilczak, Hanna Romanowicz

**Affiliations:** 1 Laboratory of Molecular Genetics, Department of Pathology, Institute of Polish Mother’s Memorial Hospital, Rzgowska 281/289, 93-338 Lodz, Poland; 2 Regional Hospital in Lodz, Lodz, Poland; 3 Department of Obstetrics and Gynaecology, Regional Hospital in Kalisz, Kalisz, Poland; 4 Cathedral of Mother’s and Child’s Health, Poznan University of Medical Sciences, Poznan, Poland; 5 Department of Clinical Psychology, Poznan University of Medical Sciences, Poznan, Poland; 6 Department of Medical Education, Poznan University of Medical Sciences, Poznan, Poland; 7 University of Computer Sciences and Skills, Lodz, Poland

**Keywords:** *XRCC2*, *XRCC3*, Triple-negative breast cancer, Gene polymorphism

## Abstract

*XRCC2* and *XRCC3* genes involved in homologous recombination repair (HRR) of DNA and in the maintenance of the genome integrity play a crucial role in protecting against mutations that lead to cancer. The aim of the present work was to evaluate associations between the risk of triple-negative breast cancer (TNBC) and polymorphisms in the genes, encoding for two key proteins of HRR: *XRCC2* Arg188His (c. 563 G>A; rs3218536, Genbank Accession Number NT 007914) and *XRCC3* Thr241Met (c. 722 C>T; rs861539, Genbank Accession Number NT 026437). The polymorphisms of the *XRCC2* and *XRCC3* were investigated by PCR–RFLP in 70 patients with TNBC and 70 age- and sex-matched non-cancer controls. In the present work, a relationship was identified between *XRCC2* Arg188His polymorphism and the incidence of triple-negative breast cancer. The 188His allele and 188His/His homozygous variant increased cancer risk. An association was confirmed between *XRCC2* Arg188His and *XRCC3* Thr241Met polymorphisms and TNBC progression, assessed by the degree of lymph node metastases and histological grades. In conclusion, *XRCC2* Arg188His and *XRCC3* Thr241Met polymorphisms may be regarded as predictive factors of triple-negative breast cancer in female population.

## Introduction

The term triple-negative breast cancer (TNBC) defines breast tumors that do not express estrogen receptors, progesterone receptor, or epidermal growth factor receptor HER2 on immunohistochemical analysis. TNBC refers to about 15–20 % of all breast cancer cases [1–5].

Molecular profiling indicated that triple-negative breast cancer represents heterogeneous subgroup of breast cancer. Triple-negative breast cancer shares histological and genetic abnormalities with basal-like subtype of breast cancer, however, this overlap is incomplete. Triple-negative breast cancer do not benefit from hormonal therapies or treatments targeted against HER2 [1–5]. Many of targeted therapeutic agents show promise in early stage studies, but their clinical performance has yet to be definitively proven.

Molecular epidemiological studies have provided the evidence that an individual’s susceptibility to precancerous lesions and cancer is modulated by both genetic and environmental factors [6, 7]. Genomic rearrangements (translocations, deletions, and duplications) are extremely frequent in breast cancer cells [8–11]. These rearrangements are believed to result from an aberrant repair of DNA double-strand breaks (DSBs).

Double-strand DNA breaks are the most dangerous DNA damage. If not repaired leads to down-regulation of transcription and various cancers development [12, 13]. DSB are repaired by two mechanisms: recombination (HR) and non-homologous end joining (NHEJ) [14, 15].

A recent study on the Caucasian population has provided the first epidemiological evidence, supporting the association between DSBs repair gene variants and breast cancer development [16].

Polymorphisms in DNA repair genes may alter the activity of the proteins and thus modulate cancer susceptibility [17].

RAD51 homolog (RecA homolog, *E. coli*) (*S. cerevisiae*) plays an important role in homologous recombination via, direct interaction with XRCC2 (X-ray repair cross-complementing group 2), XRCC3 (X-ray repair cross-complementing group 3), BRCA1 (breast cancer-1), BRCA2 (breast cancer-2), and other DNA repair proteins, to form a complex essential for repair of double-strand breaks and DNA cross-links (especially XRCC2 and XRCC3) and for the maintenance of chromosome stability [18–20].

RAD51 is involved in homologous recombination and repair of double-strand breaks in DNA and DNA cross-links and for the maintenance of chromosome stability. *RAD51* gene is highly polymorphic in nature. In the literature, many reports confirm the significance *RAD51* gene G135C polymorphism (c. 98 G>C; rs1801320; Genbank Accession Number NT 010194), regarding the risk of breast carcinoma [21–24].

*XRCC2* Arg188His polymorphism (c. 563 G>A; rs3218536, Genbank Accession Number NT 007914) may limit effect on gene activity, although it can modify the breast cancer risk in female patient with low levels of plasma α-carotene or plasma folate [16, 25].

The C722T substitution is the most thoroughly analyzed polymorphism in the *XRCC3* gene (c. 722 C>T; rs861539, Genbank Accession Number NT 026437). Although the functional relevance of *XRCC3* Thr241Met variation is unknown, some studies have reported that the 722T/T genotype is associated with increased risk of breast cancer [26–28].

In the present study, the association between the Arg188His polymorphism of *XRCC2* gene and Thr241Met polymorphism of *XRCC3* gene and triple-negative breast cancer in the population of Polish women was investigated.

## Materials and methods

### Patients

In the reported study, paraffin-embedded tumor tissue was collected from 70 women with triple-negative breast carcinoma, treated at the Department of Oncology, Institute of Polish Mother’s Memorial Hospital, Lodz, Poland. The age of the patients ranged from 36 to 68 years (the mean age 46.2 ± 10.12). No distant metastases were found in any of the patients at the time of treatment onset. The median follow-up of patients at the time of analysis was 38 months (the range 2–70 months). The average tumor size was 20 mm (the range 17–32 mm). All the tumors were graded by a method, based on the criteria of Scarff–Bloom–Richardson. The demographic data and the pathologic features of the patients are summarized in Table 1. Samples from age-matched, cancer-free women (*n* = 70) served as control (the mean age 45.41 ± 18.21). Control samples consisted of DNA extracted from normal breast tissue. Normal breast specimens were obtained from patients who had undergone biopsy for benign lesions. An appropriate ethical approval was obtained from the Ethics Committee of the Institute of Polish Mother’s Memorial Hospital, Lodz, Poland.

**Table 1:** Pathologic features of triple-negative breast cancer patients

Triple-negative breast cancer	Patients (*n* = 70)	
	*n*	(%)
Scarff–bloom–Richardson stage		
I	20	29
II	45	64
III	5	7
Tumor size grade		
T1	8	11
T2	40	57
T3	18	26
T4	4	6
Lymph node status		
N0	32	46
N1	12	17
N2	14	20
N3	7	10
N4	5	7

The breast tissue samples (cancerous and non-cancerous) were fixed routinely in formaldehyde, embedded in paraffin, cut into thin slices, and stained with hematoxylin/eosin for pathological examination. DNA for analysis was obtained from an archival pathological paraffin-embedded tumor and non-cancerous breast samples which were deparaffinized in xylene and rehydrated in ethanol and distilled water. In order to ensure that the chosen histological material is representative for cancerous and non-cancerous tissue, every tissue sample qualified for DNA extraction was initially checked by a pathologist. DNA was extracted from material using commercially available QIAmp Kit (Qiagen GmbH, Hilden, Germany) DNA purification kit according to manufacturer’s instruction.

### Genotyping

Polymorphism of *XRCC2* and *XRCC3* gene was determined by PCR–RFLP (polymerase chain reaction–restriction fragment length polymorphism), using the appropriate primers (Table 2).

**Table 2:** Primer sequences for polymerase chain reaction (PCR) analysis

	Primer sequence	PCR fragment length (bp)
*XRCC2*	forward 5′-TGTAGTCACCCATCTCTCTGC-3′	290
	reverse 5′-AGTTGCTGCCATGCCTTACA-3′	
*XRCC3*	forward 5′-GCCTGGTGGTCATCGACTC-3′	552
	reverse 5′-ACAGGGCTCTGGAAGGCACTGCTCAGCTCACGCACC-3′	

### Determination of XRCC2 genotype

The 25 μL PCR mixture contained 100 ng of DNA, 12.5 pmol of each primer, 0.2 mmol/l of dNTPs, 2 mmol/l of MgCl_2,_ and 1 U of Taq DNA polymerase. Thermal cycling conditions were as follows: initial denaturation step at 94 °C, 30 cycles at 94 °C for 30 s, and 30 s at 60 °C annealing temperature, and at 72 °C for 1 min. The final extension was performed at 72 °C for 7 min. The 290 bp amplified product was digested overnight with 1 U of *Hpn*I (New England Biolabs, Ipswich, MA, USA) at 37 °C. The wild-type allele Arg was identified by the presence of single band of 290 bp, while the mutant allele His was represented by 148 and 142 bp bands.

### Determination of XRCC3 genotype

*XRCC3* gene polymorphism was determined by PCR–RFLP, using codon 241 primers. The 25 μL PCR mixture contained 100 ng of DNA, 12.5 pmol of each primer, 0.2 mmol/l of dNTPs, 2 mmol/l of MgCl_2_, and 1 U of Taq DNA polymerase. Thermal cycling conditions were the following: 94 °C for 60 s, 56 °C for 30 s, and then 72 °C for 40 s, repeated in 30 cycles. The 552 bp amplified product was digested overnight with 5 U of *Nla*III (New England Biolabs, Ipswich, MA, USA) at 37 °C. The wild-type allele Thr was identified by the presence of two 239 and 313 bp bands, while the mutant allele Met was represented by 105, 208, and 239 bp bands.

### Statistical analysis

Genotype frequency deviations were assessed for each polymorphism, comparing Hardy–Weinberg equilibrium values with control values by the standard Chi square test. Genotype frequencies in the study cases and the controls were compared by the Chi square test. Genotype specific risks were estimated as odds ratios (ORs) with associated 95 % intervals (CIs) by unconditional logistic regression. *p* values <0.05 were considered significant. All the statistical analyses were performed, using the STATISTICA 6.0 software (Statsoft, Tulsa, OK, USA).

## Results

The genotype frequency of the *XRCC2* Arg188His polymorphism in the TNBC patients and controls is summarized in Table 3. It can be seen from the Table that there are significant differences in the frequency of genotypes (*p* < 0.05) between the two investigated groups. A weak association was observed between triple-negative breast carcinoma occurrence and the presence of at least one 188His allele. A stronger association was observed for 188His/His than for 188Arg/His heterozygous variant. In case of the Arg188His polymorphism of *XRCC2* gene, the distribution of the genotypes in the patients differed significantly from one expected from the Hardy–Weinberg equilibrium (*p* < 0.05).

**Table 3:** Distribution of 188Arg/Arg, 188Arg/His, and 188His/His genotypes and frequencies of the Arg and His alleles in patients with triple-negative breast cancer and controls

*XRCC2* Arg188His	TNBC patients (*n* = 70)		Controls (*n* = 70)		OR (95 % CI)^a^	*p* ^b^
	Number	(%)	Number	(%)		
188Arg/Arg	12	17	18	26	1.00 Ref	
188Arg/His	8	12	40	57	**0.30 (0.10–0.86)**	0.042
188His/His	50	71	12	17	**6.25 (2.38–16.39)**	0.0003
188Arg	32	23	76	54	1.00 Ref	
188His	108	77	64	46	**4.00 (2.39–6.71)**	<.0001

Data in boldface are statistically significant (*p* < 0.05)

^a^Crude odds ratio (OR), 95 % CI = confidence interval at 95 %

^b^Chi square

No statistically significant differences were observed in genotype frequencies of *XRCC3* Thr241Met polymorphism between the control group and the TNBC patients (see Table 4). Among the patients, all genotype distributions did not differ significantly (*p* > 0.05) from those expected by the Hardy–Weinberg equilibrium.

**Table 4:** Distribution of 241Thr/Thr, 241Thr/Met, and 241Met/Met genotypes and frequencies of the Thr and Met alleles in patients with triple-negative breast cancer and controls

*XRCC3* Thr241Met	TNBC patients (*n* = 70)		Controls (*n* = 70)		OR (95 % CI)^a^	*p* ^b^
	Number	(%)	Number	(%)		
241Thr/Thr	19	27	15	21	1.00 Ref	
241Thr/Met	35	49	35	50	0.78 (0.34–1.79)	0.718
241Met/Met	16	23	20	29	0.63 (0.24–1.62)	0.475
241Thr	73	52	65	46	1.00 Ref	
241Met	67	48	75	54	0.79 (0.49–1.27)	0.402

^a^Crude odds ratio (OR), 95 % CI = confidence interval at 95 %

^b^Chi square

Histological grading was related to *XRCC2* Arg188His and the *XRCC3* Thr241Met polymorphisms. Histological stages were evaluated in all the cases (*n* = 70). There were as follows: stage I—20 cases, stage II—45 cases, and stage III—5 cases. Stages II and III were accounted together for statistical analysis (see Table 5). Some correlation was observed between the *XRCC2*-Arg188His and *XRCC3*-Thr241Met polymorphisms and triple-negative breast cancer invasiveness. An increase was observed, regarding 188Arg/His heterozygotes frequency (OR 2.45; 95 % CI 0.66–9.02, *p* = 0.289) and 241Thr/Met heterozygotes (OR 2.50; 95 % CI 0.68–9.11, *p* = 0.267) in stage I patients, according to Scarff–Bloom–Richardson classification. That increase was, however, not statistically significant.

**Table 5:** Dependence of genotypes and frequencies of *XRCC2* and *XRCC3* gene polymorphism alleles on tumor stage in triple-negative breast cancer patients^a^

Stage^b^	Triple-negative breast cancer patients			
	I (*n* = 20)	II + III (*n* = 50)	OR (95 % CI)^c^	*p* ^d^
*XRCC2* Arg188His				
188Arg/Arg	4 (20)	14 (28)	1.00 Ref	
188Arg/His	14 (70)	20 (40)	2.45 (0.66–9.02)	0.289
188His/His	2 (10)	16 (32)	1.09 (0.29–4.08)	0.588
188Arg	22 (55)	48 (48)	1.00 Ref	
188His	18 (45)	52 (52)	0.76 (0.36–1.57)	0.571
*XRCC3* Thr241Met				
241Thr/Thr	4 (20)	15 (30)	1.00 Ref	
241Thr/Met	14 (70)	21 (42)	2.50 (0.68–9.11)	0.267
241Met/Met	2 (10)	14 (28)	0.53 (0.08–3.39)	0.417
241Thr	22 (55)	51 (51)	1.00 Ref	
241Met	18 (45)	49 (49)	0.85 (0.40–1.77)	0.806

^a^ *n* = 70

^b^according to scarff–bloom–Richardson criteria

^c^crude odds ratio (OR), 95 % CI = confidence interval at 95 %

^d^chi square

Table 6 shows the distribution of genotypes and the frequency of alleles in patients with (N+) and without (N−) lymph node metastases. A tendency for a decreased risk of breast cancer was observed with the occurrence of 188His/His genotype and 188His allele of *XRCC2* and 241Met/Met genotype and 241Met allele of *XRCC3* polymorphism. That decrease was, however, not statistically significant (*p* > 0.05) (see Table 6). There were no differences either in the distribution of genotypes or the frequency of alleles in the group of patients with different tumor size (Table 6).

**Table 6:** *XRCC2* and *XRCC3* gene polymorphism and triple-negative breast cancer progression^a^

	TNBC patients (*n* = 70)			TNBC patients (*n* = 70)		
	Tumor size			Node status		
	T3 + T4 *N* = 22	T1 + T2 *N* = 48	OR (95 % CI)^a^	N+ (*n* = 38)	N− (*n* = 32)	OR (95 % CI)^b^
*XRCC2* Arg188His						
188Arg/Arg	8 (36)	17 (35)	1.00 Ref	12 (32)	10 (31)	1.00 Ref
188Arg/His	8 (36)	18 (38)	2.51 (0.57–11.1)	16 (42)	11 (34)	1.21 (0.38–3.78)
188His/His	6 (28)	13 (27)	2.61 (0.54–12.3)	10 (26)	11 (34)	0.75 (0.22–2.51)
188Arg	24 (55)	52 (54)	1.00 Ref	40 (53)	31 (48)	1.00 Ref
188His	20 (45)	44 (46)	0.98 (0.48–2.01)	36 (47)	33 (52)	0.84 (0.43–1.64)
*XRCC3* Thr241Met						
241Thr/Thr	7 (32)	17 (35)	1.00 Ref	13 (35)	10 (31)	1.00 Ref
241Thr/Met	9 (41)	19 (40)	1.15 (0.35–3.76)	15 (39)	10 (31)	1.15 (0.36–3.64)
241Met/Met	6 (27)	12 (25)	1.21 (0.32–4.53)	10 (26)	12 (38)	0.64 (0.19–2.07)
241Thr	23 (52)	53 (55)	1.00 Ref	41 (54)	30 (47)	1.00 Ref
241Met	21 (48)	43 (45)	1.12 (0.55–2.30)	35 (46)	34 (53)	0.75 (0.38–1.46)

^a^T2 versus T3 + T4

^b^N− (node negative) versus N+ (node positive)

## Discussion

According to our data, it is the first time that polymorphisms in *XRCC2* and *XRCC3* genes involved in the DNA repair pathway were analyzed in the population of Polish women with TNBC. The combined effect of *XRCC2* and *XRCC3* polymorphisms on TNBC occurrence was not investigated before. The study was performed on an ethnically homogenous population, which may improve our knowledge, regarding to what an extent the genotype–phenotype relationship variations are population-related.

The polymorphisms, chosen for the study, had previously been shown to have functional significance and to be responsible factors for low DNA repair capacity phenotype, characteristic for patients with cancer including those with breast carcinoma [20].

The genes involved in DNA repair and in the maintenance of genome integrity play a crucial role in providing protection against mutations that may lead to cancer [29].

XRCC2 and XRCC3 proteins are structurally and functionally related to RAD51, which plays an important role in the homologous recombination, the process being frequently involved in cancer transformation [30].

*RAD51*, *XRCC2,* and *XRCC3* gene are highly polymorphic. A single nucleotide polymorphism, 135G/C, has been identified in the 5′ untranslated region of the *RAD51* gene and has been shown to influence gene transcription activity [31]. As it was mentioned in the Introduction above, the reports on the relationship between *RAD51* G135C polymorphism and breast cancer incidence are suggest that the *RAD51* 135C variant allele was associated with an increased risk of female breast cancer [22, 23, 32, 33].

By contrast, Brooks et al. [34] showed that *RAD51* gene variants were found to be not associated with breast cancer risk.

Other studies have shown that the *RAD51* 135C variant allele was associated with an increased risk of female breast cancer [35–37].

135C/C genotype may be associated with an elevated tumor risk among the European populations, regarding sporadic breast cancer [36]. Similar results were obtained in the Polish population [38].

In our earlier study, *RAD51* 135C allele variant was associated with an elevated risk of triple-negative breast cancer in the Polish women [39].

It is possible that the presence of C allele remains in a linkage disequilibrium with another, so far unknown, mutation located outside the coding region in the *RAD51* gene, which may be important, regarding RAD51 concentrations in plasma.

In the presented study, *XRCC2* Arg188His genotype was associated with an elevated risk of triple-negative breast cancer in the Polish population. There was a 6.25-fold increased risk of TNBC for the individuals, carrying *XRCC2*-188His/His genotype, compared with subjects carrying *XRCC2*-188Arg/Arg, 188Arg/His genotype, respectively. *XRCC2* Arg188His polymorphism was not related, either to tumor size or cancer type or grade.

In the reported study, the Arg188His polymorphism of *XRCC2* gene and Thr241Met of *XRCC3* were correlated with breast carcinoma progression. Arg188His and Thr241Met heterozygote were associated with an increased risk of stage I breast cancer.

However, other literature data were also found [40–42]. No significant associations were observed between the Thr241Met and breast cancer in Iowa and Cypriot women (40, 43).

In the Polish population, Thr241Met genotype of *XRCC3* polymorphism increased the risk of breast cancer development [41, 42, 44].

Similar to our observation, the recent reports demonstrate that *XRCC3* Thr241Met allele seems associated with an elevated breast cancer risk in non-Chinese subjects (28).

The role of position 188 in the aminoacid chain for XRCC2 protein functionality is still unknown. The several data suggest that *XRCC2* Arg188His polymorphism is not directly associated with breast cancer risk [45, 46].

In conclusion, the reported study is another evidence for the significance of Thr241Met and Arg188His genotype in breast carcinoma staging.

The obtained data show that Arg188His and Thr241Met polymorphisms of *XRCC2/3* genes may be associated with the risk of triple-negative breast carcinoma occurrence. On the other hand, a protective effect was observed of all the polymorphisms in the patients without (N-) lymph node metastasis. The obtained data suggest that the reported study may be the first observation of the polymorphisms in *XRCC2* and *XRCC3* genes, involved in the DNA repair pathway, to be associated with triple-negative breast carcinoma risk in the population of Polish women.

Finally, it is postulated that these polymorphisms may be used as predictive factors for TNBC in the Polish female population. Further studies, conducted on a larger group, are suggested to clarify this point.
